# Reversibility of hAT-MSCs phenotypic and metabolic changes after exposure to and withdrawal from HCC-conditioned medium through regulation of the ROS/MAPK/HIF-1α signaling pathway

**DOI:** 10.1186/s13287-020-02010-0

**Published:** 2020-11-27

**Authors:** Chenyang Wang, Jie Hu, Zheng Chen, Yifan Wang, Sinan Lu, Yuan Zhang, Yufeng Li, Yucheng Xiang, Yutian Ji, Cheng Zeng, Yuan Ding, Weilin Wang

**Affiliations:** 1 Department of Hepatobiliary and Pancreatic Surgery, The Second Affiliated Hospital, Zhejiang University School of Medicine, No. 88 Jiefang Road, Hangzhou, 310009 Zhejiang China; 2 Key Laboratory of Precision Diagnosis and Treatment for Hepatobiliary and Pancreatic Tumor of Zhejiang Province, No. 88 Jiefang Road, Hangzhou, 310009 Zhejiang China; 3 Department of Plastic Surgery, The First Affiliated Hospital, Zhejiang University School of Medicine, No. 79 Qingchun Road, Hangzhou, 310003 Zhejiang China; 4 Department of Hepatobiliary and Pancreatic Surgery, The First Affiliated Hospital, Zhejiang University School of Medicine, No. 79 Qingchun Road, Hangzhou, 310003 Zhejiang China; 5 Research Center of Diagnosis and Treatment Technology for Hepatocellular Carcinoma of Zhejiang Province, No. 88 Jiefang Road, Hangzhou, 310009 Zhejiang China; 6 Clinical Medicine Innovation Center of Precision Diagnosis and Treatment for Hepatobiliary and Pancreatic Diseases of Zhejiang University, No. 88 Jiefang Road, Hangzhou, 310009 Zhejiang China; 7 Clinical Research Center of Hepatobiliary and Pancreatic Diseases of Zhejiang Province, No. 88 Jiefang Road, Hangzhou, 310009 Zhejiang China

**Keywords:** Hepatocellular carcinoma, Conditioned medium, Human adipose tissue-derived mesenchymal stem cells, Cell phenotype, Glucose metabolism

## Abstract

**Background:**

Mesenchymal stem cells (MSCs) play an important role in tumor progression; concomitantly, MSCs also undergo profound changes in the tumor microenvironment (TME). These changes can directly impact the application and efficacy of MSC-based anti-tumor therapy. However, few studies have focused on the regulation of MSC fate in TME, which will limit the progress of MSC-based anti-tumor therapy. Herein, we investigated the effects of conditioned medium from human hepatocellular carcinoma cells (HCC-CM) on the phenotype and glucose metabolism of human adipose tissue-derived MSCs (hAT-MSCs).

**Methods:**

The passage 2 (P2) to passage 3 (P3) hAT-MSCs were exposed to conditioned medium from Hep3B, Huh7 and HCCLM3 cells for 4–8 weeks in vitro. Then, immunofluorescent, CCK-8 assay, EdU assay, Transwell assay, and flow cytometry were used to assess the alterations in cell phenotype in terms of cell morphology, secretory profiles, proliferation, migration, invasion, cell cycle, and apoptosis. In addition, glucose metabolism was evaluated by related kits. Next, the treated hAT-MSCs were subjected to withdrawal from HCC-CM for 2–4 weeks, and alterations in phenotype and glucose metabolism were reevaluated. Finally, the molecular mechanism was clarified by Western blotting.

**Results:**

The results revealed that after exposure to HCC-CM, hAT-MSCs developed a stellate-shaped morphology. In association with cytoskeleton remodeling, hAT-MSCs showed enhanced capacities for migration and invasion, while cell proliferation was inhibited by regulating the cell cycle by downregulating cyclins and cyclin-dependent kinases and activating the mitochondrial apoptosis pathway. In terms of glucose metabolism, our results showed mitochondrial dysfunction and elevated glycolysis of hAT-MSCs. However, interestingly, when the treated hAT-MSCs were subjected to withdrawal from HCC-CM, the alterations in phenotype and glucose metabolism could be reversed, but secretory phenotype and tumor-promoting properties appear to be permanent. Further studies showed that these changes in hAT-MSCs may be regulated by the ROS/MAPK/HIF-1α signaling pathway.

**Conclusion:**

Taken together, the effects of long-term HCC-CM treatment on phenotype and glucose metabolism in hAT-MSCs are modest and largely reversible after withdrawal, but HCC-CM endow hAT-MSCs with permanent secretory phenotype and tumor-promoting properties. This is the first report on the reversal of phenotype and glucose metabolism in tumor-associated MSCs (TA-MSCs), it is anticipated that new insights into TA-MSCs will lead to the development of novel strategies for MSC-based anti-tumor therapy.

**Supplementary Information:**

The online version contains supplementary material available at 10.1186/s13287-020-02010-0.

## Background

It is increasingly evident that the initiation and progression of tumors are not only determined by the genetic or epigenetic changes of tumor cells but also by the regulation of TME [1]. Cancer-associated fibroblasts (CAFs), the activated phenotype of fibroblasts within tumors, are one of the most important components of TME [2]. CAFs can be highly heterogeneous, with distinct expression patterns and multiple sources [3], and MSCs are one of the main sources [4]. MSCs have been reported to migrate to tumors, and in response to signals from growing tumors, MSCs continuously remodel the tumor niche, therefore profoundly affecting tumor growth and metastasis [5]. Meanwhile, under the education of tumor cells, MSCs evolve into TA-MSCs, obtain expression of α-smooth muscle actin (α-SMA) and Vimentin then be stellate in shape [6]. In this process, the fate of MSCs will also undergo profound changes, including cell phenotype, metabolic pattern and cell function. However, few studies have focused on the regulation of MSC fate in TME, which is not conducive to fully understanding TME.

As a kind of non-hematopoietic stem cell, MSCs have the ability to self-renew and to differentiate into various cell types, including adipocytes, osteoblasts and chondrocytes. Due to the characteristics of ‘tumor homing’ and ‘immune privilege’, MSCs have recently been considered as viable tools for anticancer approaches [7]. However, with further preclinical study, studies have suggested that MSCs could undergo malignant transformation in TME [8]. There have also been reports that the degree of engraftment is generally low and transient [9]. In addition, MSCs have a special temporal-spatial pattern in tumors: MSCs can be concentrated inside the tumor in a short time and will redistribute to the tumor boundary after a certain period of time [10, 11]. Thus, the problems of safety, engraftment efficiency and redistribution also restrict the progress of MSC-based anti-tumor therapy.

In addition, cellular energy derives mainly from glucose metabolism, and cellular fate correlates closely with energy status. Tumor cells are known for their altered glucose metabolism characteristic, which is the metabolic shift to aerobic glycolysis, regardless of oxygen availability (the ‘Warburg Effect’) [12]. Recently, some studies have found that CAFs can also have a similar effect [13]. Further analysis showed that tumor cells induced oxidative stress in CAFs, which then led to mitochondrial dysfunction and acted as a ‘metabolic’ motor to drive aerobic glycolysis. As a consequence, CAFs provided nutrients such as lactate and pyruvate to simulate mitochondrial biogenesis and oxidative metabolism in adjacent tumor cells (the “Reverse Warburg Effect”) [14]. Nevertheless, almost all research has focused on CAFs, and none of the reports were detailed regarding MSCs. Therefore, exploring the changes in glucose metabolism of MSCs is conducive to gain a more complete understanding of cell fate regulation, which has profound practical value.

In summary, we believe that clarifying the regulation of MSC fate in TME is of great practical value for understanding TME and solving the current dilemma of MSC-based anti-tumor therapy. Only when we figure out the cell fate regulation during the transformation of MSCs into TA-MSCs, especially the changes in phenotype and glucose metabolism, can we understand the meaning of the corresponding functional changes, which will enable targeted engineering MSCs to make them more suitable for the role of drug-carrying tools.

Herein, we investigated the effects of conditioned medium from human hepatocellular carcinoma cells on the phenotype and glucose metabolism of human adipose tissue-derived MSCs (hAT-MSCs) and its molecular mechanism to develop novel strategies for MSC-based anti-tumor therapy.

## Materials and methods

### Identification of hAT-MSCs

The hAT-MSC cell line was a gift from Dr. Zhenhua Hu (The Fourth Affiliated Hospital, College of Medicine, Zhejiang University, China). To further identify the hAT-MSCs, we conducted flow cytometric analysis and in vitro differentiation assays. To clarify the surface markers of MSCs, we utilized a Human Mesenchymal Stem Cell Multi-Color Flow Kit (R&D Systems, Minneapolis, MN, USA) to label CD45, CD90, CD105 and CD146. hAT-MSCs were detached by trypsin and resuspended in PBS and then incubated in medium with antibodies for 30 mins. After washing with PBS, flow cytometric analysis was performed. To assess the differentiation potential of hAT-MSCs towards osteoblasts, adipocytes and chondroblasts, we induced the hAT-MSCs by osteogenic, adipogenic and chondrogenic media (Cyagen, Guangzhou, China), respectively. Cells were stained after 3–4 weeks of incubation. The hAT-MSCs were stained with Alizarin Red S (Sigma-Aldrich Co., St Louis, MO, USA), Oil Red O solution (Sigma-Aldrich Co., St Louis, MO, USA) and Alcian blue (Sigma-Aldrich Co., St Louis, MO, USA).

### Cell culture

The HCC cell lines Hep3B and Huh7 were purchased from Shanghai Cell Bank, Chinese Academy of Sciences, HCCLM3 was established at our laboratory. Cells were cultured in DMEM (Biological Industries, Kibbutz Beit-Haemek, Israel) supplemented with 10% foetal bovine serum (Biological Industries, Kibbutz Beit-Haemek, Israel), penicillin (100 units/mL, Gibco, Carlsbad, CA, USA) and streptomycin (100 *μ*g/mL, Gibco). All cells were incubated under a humidified atmosphere at 37 °C, 5% CO_2_. Hep3B (4 × 10^5^), Huh7 (5 × 10^5^) and HCCLM3 (6 × 10^5^) were seeded into a 10-cm dish and cultured for 2 days. Then the HCC-conditioned medium was collected and filtered through a 0.22-*μ*m filter.

The hAT-MSCs from passage 2 (P2) to passage 3 (P3) were divided into 4 groups. Three of the groups were experimental groups that were cultured in Hep3B-conditioned medium (3B-CM), Huh7-conditioned medium (Huh7-CM) and HCCLM3-conditioned medium (LM3-CM) for 4–8 weeks and were marked as treated hAT-MSCs, with the medium replaced every 2 days. The other group was treated with normal medium as a control.

For phenotypic and metabolic reversal experiments, the treated hAT-MSCs were replaced in normal medium for another 2–4 weeks and were marked as R-3B-CM, R-Huh7-CM and R-LM3-CM, respectively.

Untreated hAT-MSCs (2 × 10^5^), treated hAT-MSCs (2 × 10^5^) and reversed hAT-MSCs (2 × 10^5^) were seeded into a 10-cm dish and cultured for 3 days. Then the MSC-conditioned medium was collected and filtered through a 0.22-*μ*m filter. Then the HCC cells were divided into 4 groups, respectively. Three of the groups were experimental groups that were cultured in untreated hAT-MSC-conditioned medium (U-MSC-CM), treated hAT-MSC-conditioned medium (T-MSC-CM) and reversed hAT-MSC-conditioned medium (R-MSC-CM) for 3 days. The other group was treated with normal medium as a control.

### Cell proliferation assays

hAT-MSCs (8 × 10^3^) in each group (untreated hAT-MSCs, treated hAT-MSCs and reversed hAT-MSCs) and HCC cells (3 × 10^3^) in each group (DMEM, U-MSC-CM, T-MSC-CM and R-MSC-CM) were seeded into 96-well plates, and cell proliferation was measured at different time-points using the Cell Counting Kit-8 (Dojindo Molecular Technologies Inc., Tokyo, Japan) for 96 h according to the manufacturer’s protocol.

We also investigated the newly synthesized DNA of hAT-MSCs in each group using the EdU assay (RiboBio, Guangzhou, China). Cells (1 × 10^4^) were seeded into 48-well plates and exposed to 50 μM 5-ethynyl-2′-deoxyuridine for 5 h at 37 °C. Then, the remaining procedures were all performed according to the manufacturer’s protocol.

### Cell cycle analysis

Flow cytometry was applied for cell cycle analysis. Approximately 2 × 10^5^ hAT-MSCs in each group were harvested and fixed in 75% ethanol for 24 h at − 20 °C, and the cells were then stained with DNA staining solution (LiankeBio, Hangzhou, China) for 30 mins to detect cell cycle distribution.

### Cell apoptosis assays

Approximately 5 × 10^5^ hAT-MSCs in each group were harvested and then detected using an Annexin V-FITC/PI Apoptosis Detection Kit (LiankeBio, Hangzhou, China) according to the manufacturer’s protocol.

### Transwell assays

The chemotaxis, migration and invasion assays were performed in Transwell chambers with 8 μm pore size (Corning, NY, USA). To measure the migratory capacity to different HCC-conditioned medium of hAT-MSCs, untreated hAT-MSCs (5 × 10^4^) in 200 μL of serum-free medium were seeded in the upper chamber, and 800 μL of HCC-conditioned medium or DMEM with 10% FBS was placed in the lower chamber. The chambers were incubated at 37 °C for 20 h. Subsequently, cells were stained with Crystal Violet Staining Solution (Beyotime, Shanghai, China). The number of cells that had migrated across the transwell membrane was counted based on the number of stained nuclei using an inverted microscope (Leica, Malvern, PA, USA).

For migration assays, hAT-MSCs in each group and HCC cells in each group (5 × 10^4^) in 200 μL of serum-free medium were seeded in the upper chamber, and 800 μL of DMEM with 10% FBS was placed in the lower chamber. The remaining procedures were the same as the chemotaxis assay.

For invasion assays, 50 μL of diluted BD Matrigel (1: 8) was added to the upper chambers and incubated at 37 °C until completely solid. Then, hAT-MSCs in each group were added to the upper chambers, and the time of incubation was extended to 72 h. The remaining procedures were the same as the chemotaxis assay.

### Immunofluorescent assays

To determine the alterations of cytoskeleton structure, hAT-MSCs in each group (2 × 10^4^) were seeded into 6-well plates. Cells were fixed in 4% paraformaldehyde (Saiguobio, Guangzhou, China) for 15 min and washed 2 times with PBS. Then, cells were permeabilized with 0.5% Triton X-100 (Solarbio, Beijing, China) for 15 mins and blocked with 5% BSA solution (Beyotime, Shanghai, China) for 1 h with gentle agitation. After blocking nonspecific binding, the cells were incubated with primary antibodies against α-SMA (1:250, Abcam, Cambridge, MA, USA) overnight at 4 °C. Then, the cells were washed for 3 times with PBS. Next, cells were stained with the FITC-labeled secondary antibody (Beyotime, Shanghai, China) and counterstained with DAPI (1:1000, 5 μg/ml, Beyotime, Shanghai, China) to visualize the nuclei. Cells were observed using a fluorescence microscope.

### Western blot analysis

Approximately 5 × 10^5^ hAT-MSCs in each group were harvested, and total proteins were then extracted from the cells by incubating in RIPA cell lysis buffer with 1% PMSF and phosphatase inhibitors (Servicebio, Wuhan, China) on ice for 30 mins. After centrifugation (14,000×g, 4 °C, 15 mins), the supernatant was collected, and the total protein contents were measured using a BCA protein assay kit (Thermo Fisher Scientific, Waltham, MA, USA). Next, proteins were separated by 4%–12% SurePAGE Bis-Tris gels (Genscript, Nanjing, China) at consistent 120 V for 60 mins. Then, the proteins were transferred to PVDF membranes at consistent 350 mA for 60 mins. After the membranes were blocked using 5% BSA solution for 2 h, the membranes were incubated overnight at 4 °C with primary antibodies against β-actin (1:2000, ab8226, Abcam), α-SMA (1:1000, ab32575, Abcam), Vimentin (1:1000, 10,366–1-AP, Proteintech), Cyclin B1 (1:1000, ab32053, Abcam), Cyclin A2 (1:1000, 18,202–1-AP, Proteintech), Cyclin D1 (1:1000, ab16663, Abcam), Cyclin E1 (1:1000, ab33911, Abcam), CDK1 (1:1000, ab18, Abcam), CDK2 (1:1000, ab32147, Abcam), CDK4 (1:1000, ab137675, Abcam), Bcl-2 (1:1000, 12,789–1-AP, Proteintech), Caspase 3 (1:500, ab13847, Abcam), Bax (1:1000, 50,599–2-Ig, Proteintech), GLUT1 (1:500, ab40084, Abcam), HK2 (1:1000, ab104836, Abcam), GPI (1:1000, 15,171–1-AP, Proteintech), GAPDH (1:2000, ab9484, Abcam), PGK1 (1:1000, ab199438, Abcam), PKM2 (1:1000, ab85555, Abcam), LDH-A (1:1000, ab101562, Abcam), LDH-B (1:1000, ab75167, Abcam), HIF-1α (1:250, ab1, Abcam), AKT (1:1000, ab179463, Abcam), JNK (1:2000, 24,164–1-AP, Proteintech), p38 (1:1000, 14,064–1-AP, Proteintech), ERK1/2 (1:1000, ab17942, Abcam), p-AKT (1:1000, ab81283, Abcam), p-JNK (1:2000, AF3318, Affinity), p-p38 (1:1000, AF4001, Affinity) and p-ERK1/2 (1:1000, ab201015, Abcam). After washing 3 times with TBST, the membranes were incubated with secondary anti-mouse or anti-rabbit antibodies (1:3000, Servicebio, Wuhan, China) for 1 h at room temperature. Detection was carried out using the ECL kit (Servicebio, Wuhan, China).

### Glucose uptake, lactate, pyruvate and ATP assays

The Glucose Uptake Colorimetric Assay Kit (BioVision, Milpitas, CA, USA), Lactate Colorimetric Assay Kit II (BioVision), Pyruvate Colorimetric Assay kit (BioVision) and ATP Colorimetric Assay Kit (BioVision) were used to determine glucose uptake and levels of lactate, pyruvate and ATP, respectively, according to the manufacturer’s protocols.

### Mitochondrial staining

To stain mitochondria, we used Mito-Tracker Green (100 nM, Beyotime, Shanghai, China) to mark mitochondrial mass and JC-1 (10 μg/ml, Beyotime, Shanghai, China) to mark mitochondrial membrane potential (MMP). hAT-MSCs in each group (2 × 10^4^) were seeded into 6-well plates. After 24 h, cells were incubated with Mito-Tracker or JC-1 staining solution for 30 mins at 37 °C. Later, cells were washed in normal medium. Cells were observed using a fluorescence microscope.

### RNA extraction and quantitative real-time PCR

Total cellular RNA was extracted using an RNA-Quick Purification Kit (Yishan Biotechnology Co., Shanghai, China) and reverse transcribed to cDNA using a HiScript RT kit (Vazyme, Nanjing, China) in accordance with the manufacturer’s protocol. RT-qPCR was performed using specific primers for the quantification of GLUT1, HK2, GPI, GAPDH, PGK1, PKM2, LDHA, IL-1β, IL-6, IL-8, TGF-β, TNF-α, CCL-2 and CCL-7 (Table 1). β-actin was used as a control. Reactions were performed using the ChamQ universal SYBR master mix (Vazyme, Nanjing, China) in the Bio-Rad CFX96 Real-Time System. mRNA expression was calculated using the 2^–ΔΔCT^ method.

**Table 1:** Primer sequences of target genes

Gene	Primer sequences
β-actin	F: AGGGGCCGGACTCGTCATACT
	R: GGCGGCACCACCATGTACCCT
GLUT1	F: CATCCCATGGTTCATCGTGGCTGAACT
	R: GAAGTAGGTGAAGATGAAGAACAGAAC
HK2	F: GCCATCCTGCAACACTTAGGGCTTGAG
	R: GTGAGGATGTAGCTTGTAGAGGGTCCC
GPI	F: TATTGTGTTCACCAAGCTCACACC
	R: TGGTAGAAGCGTCGTGAGAGGTC
GAPDH	F: TTCCGTGTCCCCACTGCCAACGT
	R: CAAAGGTGGAGGAGTGGGTGTCGC
PGK1	F: ATGTCGCTTTCTAACAAGCTGA
	R: GCGGAGGTTCTCCAGCA
PKM2	F: GCCCGTGAGGCAGAGGCTGC
	R: TGGTGAGGACGATTATGGCCC
LDHA	F: ATGGCAACTCTAAAGGATCA
	R: GCAACTTGCAGTTCGGGC
IL-1β	F: GTGAGTAGGAGAGGTGAGAGAGG
	R: ATAGCCTGGACTTTCCTGTTGTC
IL-6	F: TACATCCTCGACGGCATCTC
	R: AGCTCTGGCTTGTTCCTCAC
IL-8	F: GCTCTGTGTGAAGGTGCAGTTT
	R: TTCTGTGTTGGCGCAGTGT
TGF-β	F: GAGGTGACCTGGCCACCATT
	R: TCCGCAAGGACCTCGGCTGG
TNF-α	F: CCGAGTGACAAGCCTGTAGC
	R: AGGAGGTTGACCTTGGTCTG
CCL-2	F: GAACCGAGAGGCTGAGACTA
	R: GCCTCTGCACTGAGATCTTC
CCL-7	F: GACAAGAAAACCCAAACTCCAAAG
	R: TCAAAACCCACCAAAATCCA

### Measurement of ROS

Intracellular ROS were measured using a Reactive Oxygen Species Assay Kit (Yeasen Biotech Co., Shanghai, China). Approximately 2 × 10^5^ hAT-MSCs in each group were harvested and incubated with 10 μM DCFH-DA Solution for 30 min at 37 °C. The remaining procedures were performed in accordance with the manufacturer’s protocol.

### Statistical analysis

Data are presented as the mean ± SD of three independent experiments. All statistical analyses were performed in GraphPad Prism version 8.0 software (GraphPad Software Inc., San Diego, CA, USA) using one-way or two-way ANOVA. For all statistical tests, the level of *p* < 0.05 was considered as significance.

## Results

### hAT-MSCs migrated to TME and evolved into TA-MSCs

**Fig. 1:**
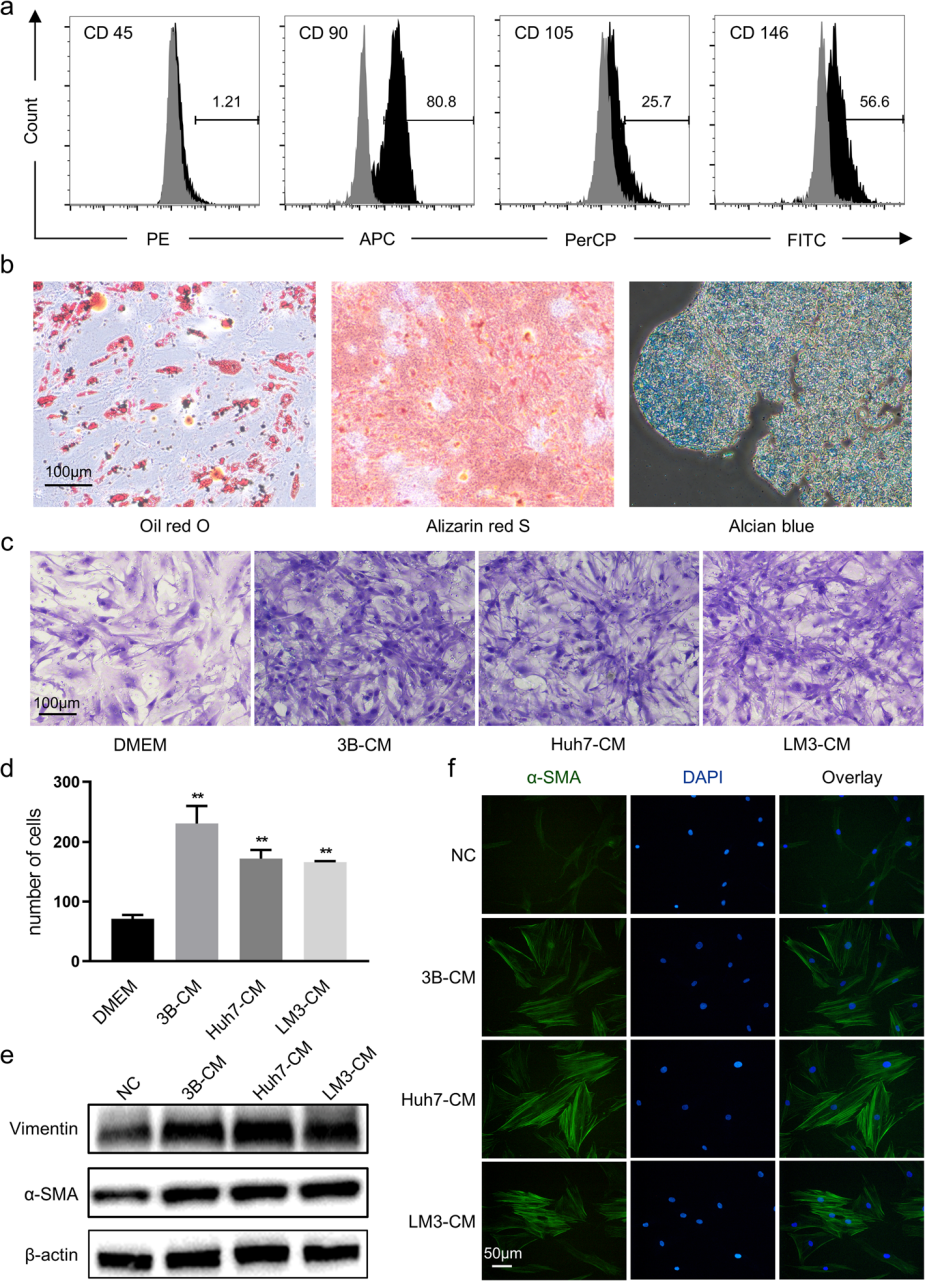
Identification results of hAT-MSCs and their characteristics. **a** Surface markers of hAT-MSCs. **b** Differentiation in vitro of hAT-MSCs. **c** & **d** Chemotaxis assays (left) and quantitative results (right, numbers of stained nuclei) of hAT-MSCs towards HCC-CM and normal medium; **e** α-SMA and Vimentin expression levels were evaluated by Western blot of hAT-MSCs after exposure to HCC-CM or normal medium; **f** α-SMA expression levels and distribution were evaluated by immunofluorescence of hAT-MSCs after exposure to HCC-CM or normal medium. **p < 0.01, vs DMEM, *n* = 2. Abbreviations: 3B-CM: Hep3B-conditioned medium, Huh7-CM: Huh7-conditioned medium, LM3-CM: HCCLM3-conditioned medium

Before analyzing the effects of HCC-CM on hAT-MSC phenotype and glucose metabolism, we first needed to characterize the obtained cells. Flow cytometry indicated that the obtained cells were positive for the MSC surface markers CD90, CD105 and CD146 and negative for the hematopoietic marker CD45 (Fig. 1a). Experiments of differentiation in vitro indicated that the obtained cells could differentiate to adipocytes, osteocytes and chondrocytes (Fig. 1b). Thus, we determined that the obtained cells were indeed hAT-MSCs.

In the next experiment, the precondition was based entirely on the tumor-homing properties of MSCs. To verify the chemotaxis capacity of untreated hAT-MSCs towards the liver cancer environment, we designed in vitro chemotaxis assays. Untreated hAT-MSCs were allowed to migrate towards HCC-CM (Hep3B, Huh7 and HCCLM3) or DMEM as a control. A significant increase in the migratory capacity of hAT-MSCs towards HCC-CM was found when compared to DMEM (Fig. 1c, d). The results suggested that hAT-MSCs were actively recruited to HCCs.

Once MSCs migrated to TME, tumor cells can ‘educate’ MSCs to evolve into TA-MSCs via paracrine interaction. As a consequence, MSCs gain expression of α-SMA and Vimentin and become stellate in shape. Therefore, we decided to study the changes in hAT-MSC properties after HCC-CM stimulation (treated hAT-MSCs). After 4–8 weeks of exposure to HCC-CM, the results of Western blot showed that the expression of α-SMA and Vimentin in hAT-MSCs increased significantly (Fig. 1e). To further investigate the effects of HCC-CM on morphological and cytoskeletal changes in hAT-MSCs, immunofluorescence assays were performed. The results showed that hAT-MSCs exhibited strong α-SMA expression following treatment with HCC-CM, in keeping with the morphology as cruciform or stellate shape (Fig. 1f). All of the above results suggested that hAT-MSCs can be truly converted into TA-MSCs after being induced by HCC-CM.

### Phenotypic changes of hAT-MSCs after exposure to HCC-CM

**Fig. 2:**
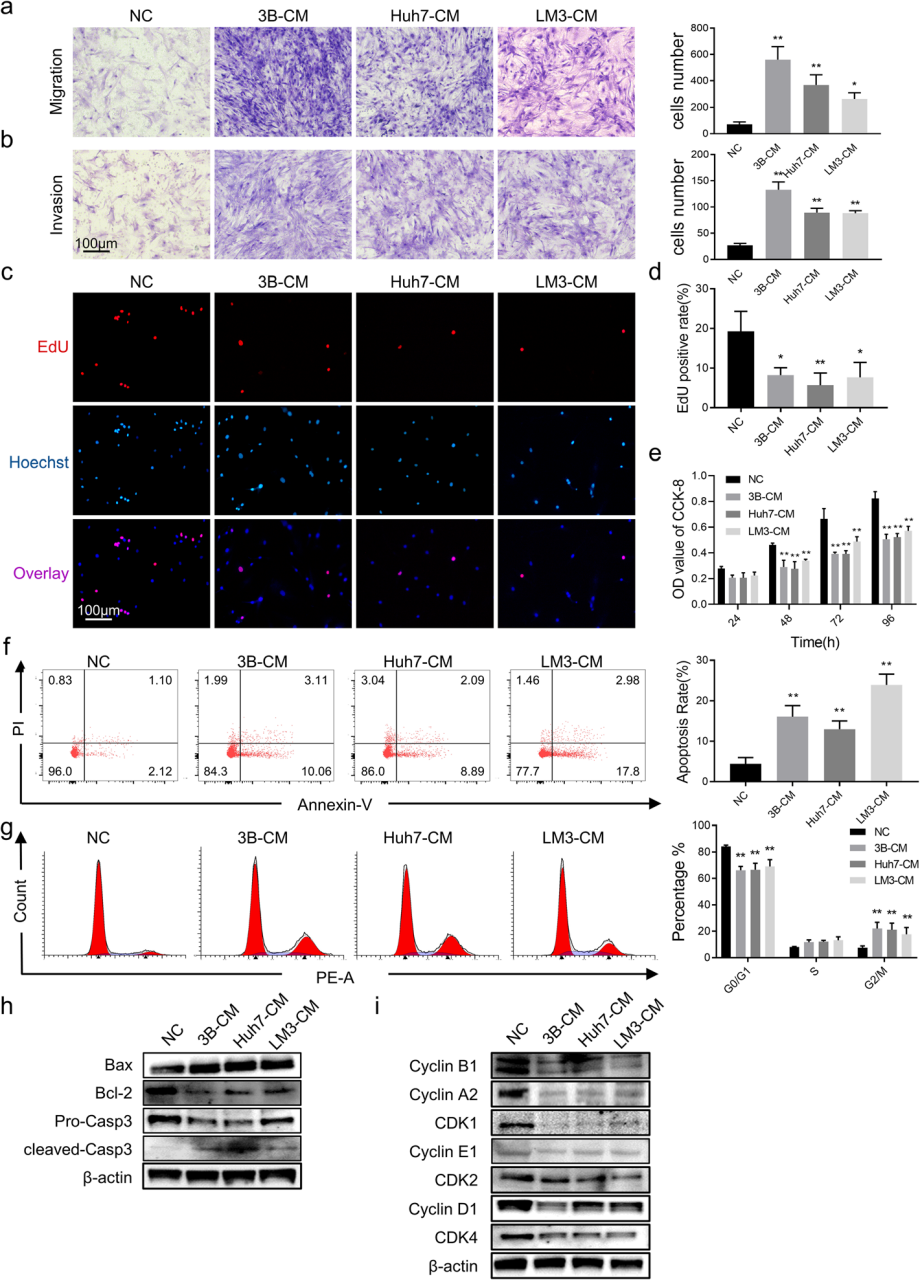
Effects of HCC-CM on cell phenotype of hAT-MSCs. (**a**) Cell migration assays (left) and quantitative results (right, numbers of stained nuclei) of hAT-MSCs after exposure to HCC-CM or normal medium; (**b**) Cell invasion assays (left) and quantitative results (right, numbers of stained nuclei) of hAT-MSCs after exposure to HCC-CM or normal medium; (c & d) EdU assays (**c**) and quantitative results (**d**) of hAT-MSCs after exposure to HCC-CM or normal medium; (**e**) Quantitative results of OD value at 570 nm in hAT-MSCs after exposure to HCC-CM or normal medium; (**f**) Representative images (left) and quantification results (right) of cell apoptosis in hAT-MSCs after exposure to HCC-CM or normal medium; (**g**) Representative images (left) and quantification results (right) of cell cycle distribution in hAT-MSCs after exposure to HCC-CM or normal medium; (**h** & **i**) The expression levels of apoptosis-related (**h**) and cell cycle-related (**i**) proteins were evaluated by Western blot of hAT-MSCs after exposure to HCC-CM or normal medium. *p < 0.05, **p < 0.01, vs NC, *n* = 2, except for CCK-8 assays (*n* = 8)

The remodeling and reorganization of the cytoskeleton can impact cellular motility. Therefore, we decided to study the migration and invasion capacities of hAT-MSCs after exposure to HCC-CM. Significant increases in the migratory and invasion capacities of treated hAT-MSCs were found (Fig. 2a, b).

Next, to evaluate the effect of HCC-CM on the proliferation of hAT-MSCs, CCK-8 assays and EdU staining were used. EdU assay results showed a significant reduction in EdU-positive cells after exposure to HCC-CM (Fig. 2c, d). In accordance with the results of EdU assays, CCK-8 assays revealed significant suppression of cell viability after HCC-CM stimuli, except for the 24-h point (Fig. 2e). In brief, proliferation of hAT-MSCs was significantly inhibited by HCC-CM.

Considering that distribution of the cell cycle and level of cell apoptosis can influence cell proliferation and viability, flow cytometry was applied to analyze hAT-MSC apoptosis and cell cycle. The results of cell apoptosis showed a markedly higher cell apoptosis rate (Annexin V +) in hAT-MSCs after exposure to HCC-CM (Fig. 2f). Regarding the cell cycle, we observed that after exposure to HCC-CM, the ratio of cells in the G0/G1 phase was significantly increased while in the G2/M phase it’s significantly reduced, which suggested that HCC-CM induced G2/M phase cell cycle arrest (Fig. 2g).

Finally, we determined the expression levels of apoptosis- and cell cycle-related proteins by Western blot. The results showed that the mitochondrial apoptosis pathway was activated in hAT-MSCs after exposure to HCC-CM. The results revealed an increase in the expression levels of apoptotic proteins (Bax and cleaved caspase-3) and a decrease in anti-apoptotic protein (Bcl-2) (Fig. 2h). Regarding cycle-related regulatory proteins, cyclins and cyclin-dependent kinases were generally downregulated, especially the expression levels of regulatory proteins related to the G2/M phase (Cyclin A2, Cyclin B1, and CDK1) (Fig. 2i).

These data demonstrate that after exposure to HCC-CM, the proliferation of hAT-MSCs was inhibited by regulating the cell cycle and activating the mitochondrial apoptosis pathway, while the migratory and invasion capacities were significantly enhanced.

### Glucose metabolic changes of hAT-MSCs after exposure to HCC-CM

**Fig. 3:**
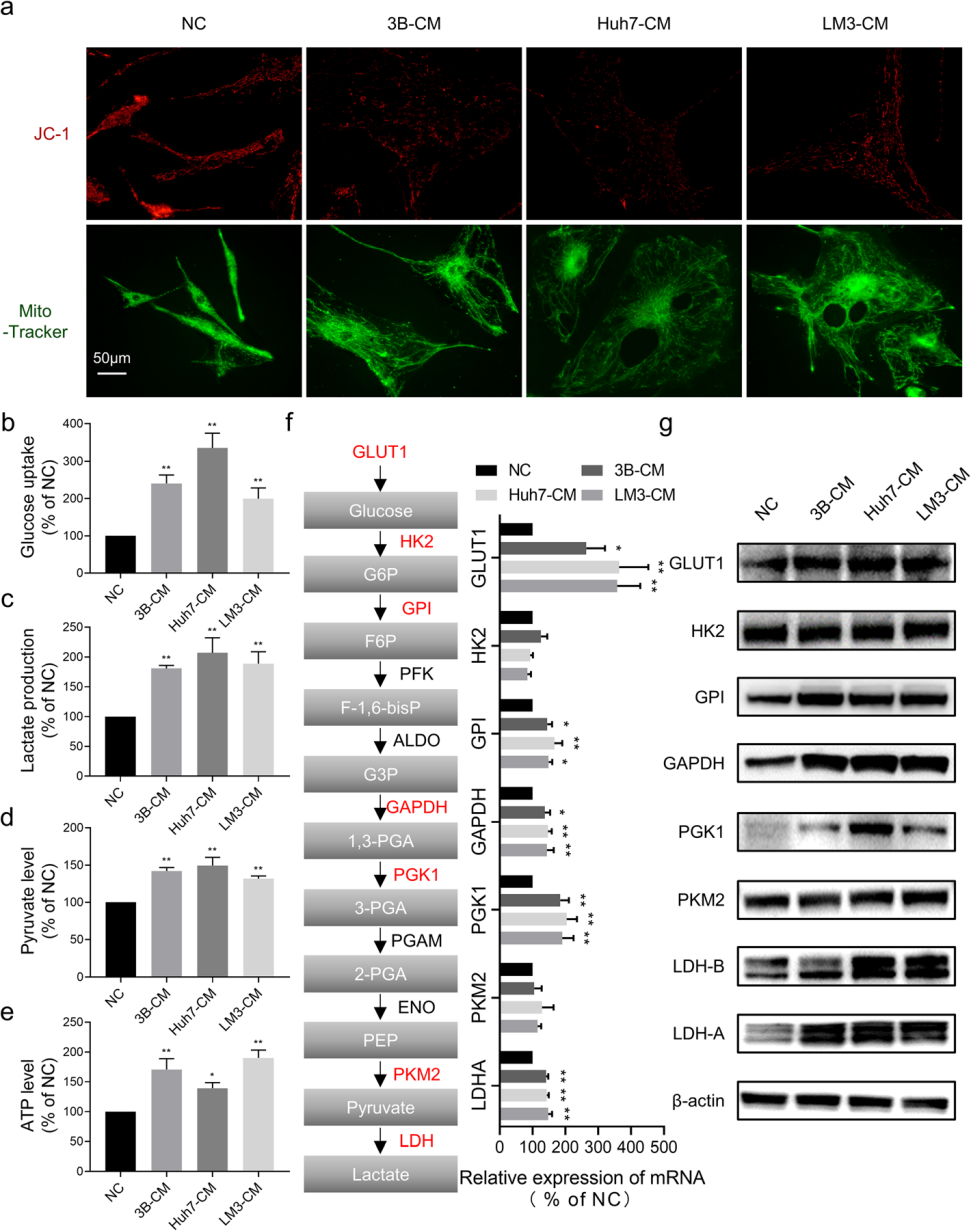
Glycolysis and mitochondrial function of hAT-MSCs after exposure to HCC-CM or normal medium. (**a**) Representative images of mitochondrial mass (Mito-Tracker, 100 nM) and MMP (JC-1, 10 μg/ml) in hAT-MSCs after exposure to HCC-CM or normal medium; (b-e) Quantification of relative glucose uptake (**b**), lactate production (**c**), pyruvate level (**d**) and ATP level (**e**) in hAT-MSCs after exposure to HCC-CM or normal medium; (**f** & **g**) Analysis of glycolytic gene expression in hAT-MSCs after exposure to HCC-CM or normal medium. qRT-PCR (**f**) and immunoblot (**g**). A schematic diagram of the glycolysis pathway is shown on the left (**f**). *p < 0.05, **p < 0.01, vs NC, *n* = 3

As we described above, the mitochondrial apoptosis pathway of hAT-MSCs was activated after exposure to HCC-CM; thus, we wondered whether the function of mitochondria could be influenced by HCC-CM. Maintenance of mitochondrial transmembrane potential is essential for normal mitochondrial function. Therefore, we used JC-1 and Mito-Tracker to stain mitochondria. The accumulation of JC-1 within the mitochondria is dependent on the electrochemical gradient, so it can be used to evaluate the MMP. However, Mito-Tracker is independent of membrane potential; thus, mitochondrial mass can be monitored with it. The staining results showed that the mitochondrial mass of hAT-MSCs was essentially unaffected after exposure to HCC-CM, while there was a marked decline in MMP (Fig. 3a). These results suggest that HCC-CM induced mitochondrial dysfunction.

Mitochondria are the cellular bioenergetic centres, and mitochondrial dysfunction will lead to a metabolic switch from oxidative phosphorylation to glycolysis. Therefore, we tested whether HCC-CM modulated the glycolytic phenotype in hAT-MSCs. The results showed significant increases in glucose uptake, pyruvate level, lactate production and ATP level of hAT-MSCs after exposure to HCC-CM (Fig. 3b-e). Moreover, after exposure to HCC-CM, hAT-MSCs showed markedly enhanced GLUT1, GPI, GAPDH, PGK1, LDHA and LDHB, but not HK2 and PKM2, at the mRNA and protein levels (Fig. 3f, g).

These data indicate glycolysis is enhanced in hAT-MSCs after exposure to HCC-CM due to mitochondrial dysfunction.

### Reversal of phenotypic changes in hAT-MSCs after withdrawal of HCC-CM treatment

Our previous study showed that the fate of hAT-MSCs had been seriously threatened after exposure to HCC-CM, while cells gained the capacities of migration and invasion. Thus, we wondered whether treated hAT-MSCs would escape from TME and reverse their fate. To verify the conjecture, we decided to culture treated hAT-MSCs in normal medium for another 2–4 weeks and to evaluate the reversibility in phenotype and glucose metabolism.

**Fig. 4:**
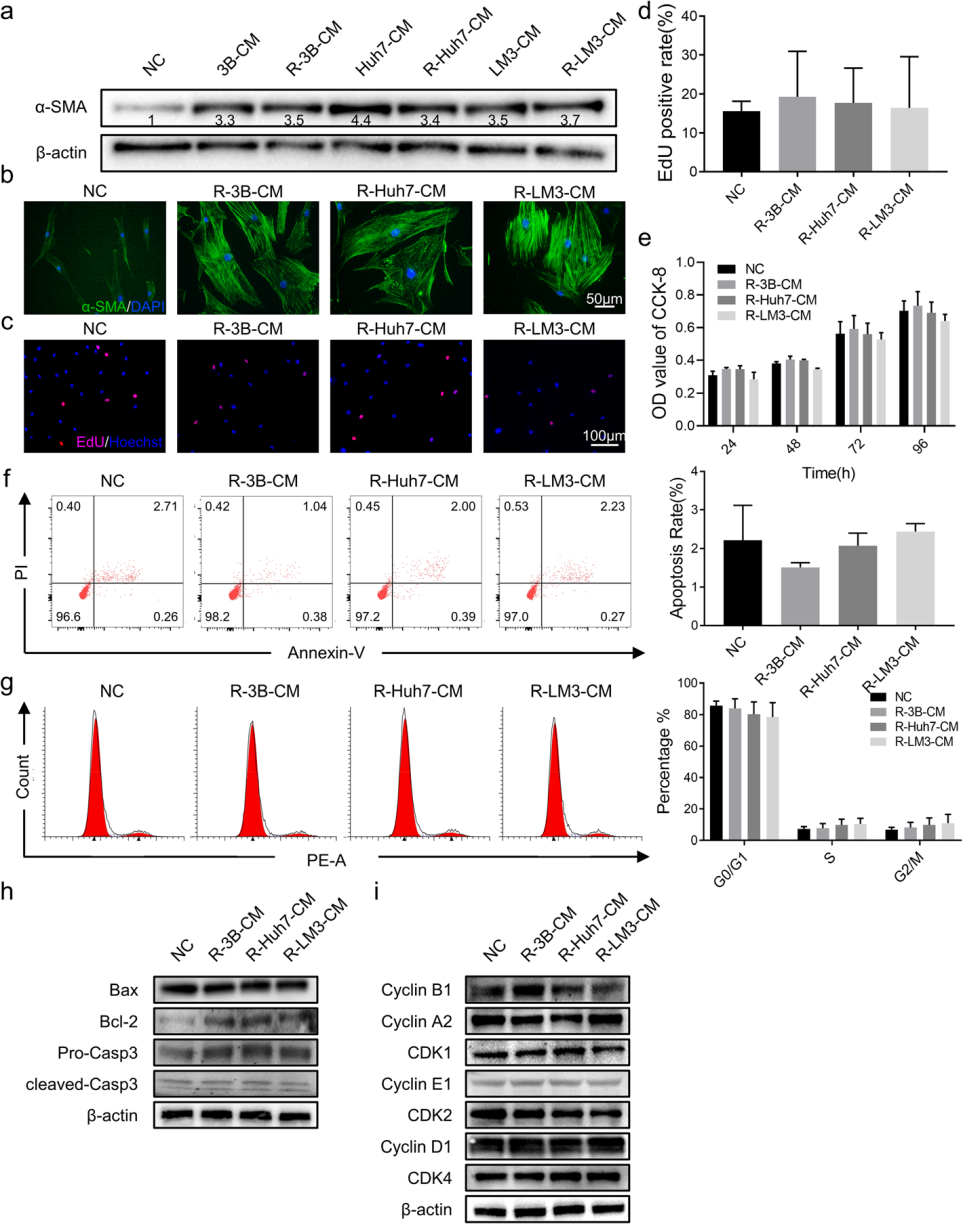
Proliferation, apoptosis and cell cycle of hAT-MSCs after withdrawal of HCC-CM treatment. **a** α-SMA expression was evaluated by Western blot of hAT-MSCs after exposure to HCC-CM and withdrawal of HCC-CM treatment; (**b**) α-SMA expression and distribution were evaluated by immunofluorescence assays of hAT-MSCs after withdrawal of HCC-CM treatment; **c**, **d** EdU assays (**c**) and quantitative results (**d**) of hAT-MSCs after withdrawal of HCC-CM treatment; **e** CCK-8 assays and quantitative results of hAT-MSCs after withdrawal of HCC-CM treatment; **f** Representative images (left) and quantification results (right) of cell apoptosis in hAT-MSCs after withdrawal of HCC-CM treatment; **g** Representative images (left) and quantification results (right) of cell cycle distribution in hAT-MSCs after withdrawal of HCC-CM treatment; **h** & **i** The expression of apoptosis-related (**h**) and cell cycle-related (**i**) proteins were evaluated by Western blot of hAT-MSCs after withdrawal of HCC-CM treatment. vs NC, *n* = 2, except for CCK-8 assays (*n* = 8). Abbreviations: R-3B-CM: reversed-Hep3B-conditioned medium, R-Huh7-CM: reversed-Huh7-conditioned medium, R-LM3-CM: reversed-HCCLM3-conditioned medium

Before verification, we needed to verify if the treated hAT-MSCs still retained the same characteristic of TA-MSCs after withdrawal of HCC-CM treatment. Western blot results indicated that hAT-MSCs maintained the high-level expression of α-SMA even after withdrawal of HCC-CM treatment (Fig. 4a). In addition, the results of immunofluorescence suggested that hAT-MSCs still maintained their cruciform or stellate shape after withdrawal of HCC-CM treatment (Fig. 4b). These findings indicate that HCC-CM can induce permanent alterations during the conversion of hAT-MSCs into TA-MSCs.

Once again, we evaluated proliferation of treated hAT-MSCs after the detachment from HCC-CM by CCK-8 assays and EdU staining. The results showed no significant difference in proliferation between the reversed hAT-MSCs and untreated hAT-MSCs (Fig. 4c-e). These findings suggest the reversal of HCC-CM-induced inhibition of proliferation in hAT-MSCs after withdrawal of HCC-CM treatment.

We then used flow cytometry to analyze reversed hAT-MSC apoptosis and cell cycle. The results showed that the apoptosis rate (Annexin V +) and cell cycle distribution were not significantly different between the groups (Fig. 4f, g). These data demonstrate that both activated apoptosis and cell cycle arrest returned to control levels after withdrawal of HCC-CM treatment.

Finally, we determined the expression levels of cell cycle- and apoptosis-related proteins by Western blot. The results revealed a slight increase in the expression of anti-apoptotic protein (Bcl-2) and no difference in apoptotic proteins (Bax and cleaved caspase-3) (Fig. 4h). Regarding cell cycle-related regulatory proteins, all cyclins and cyclin-dependent kinases were restored, consistent with the untreated hAT-MSCs (Fig. 4i).

These data indicate that HCC-CM-induced inhibition of proliferation, activation of the mitochondrial apoptosis pathway and cell cycle arrest in hAT-MSCs can be reversed and restored to normal by withdrawal of HCC-CM treatment.

### Reversal of metabolic changes in hAT-MSCs after withdrawal of HCC-CM treatment

**Fig. 5:**
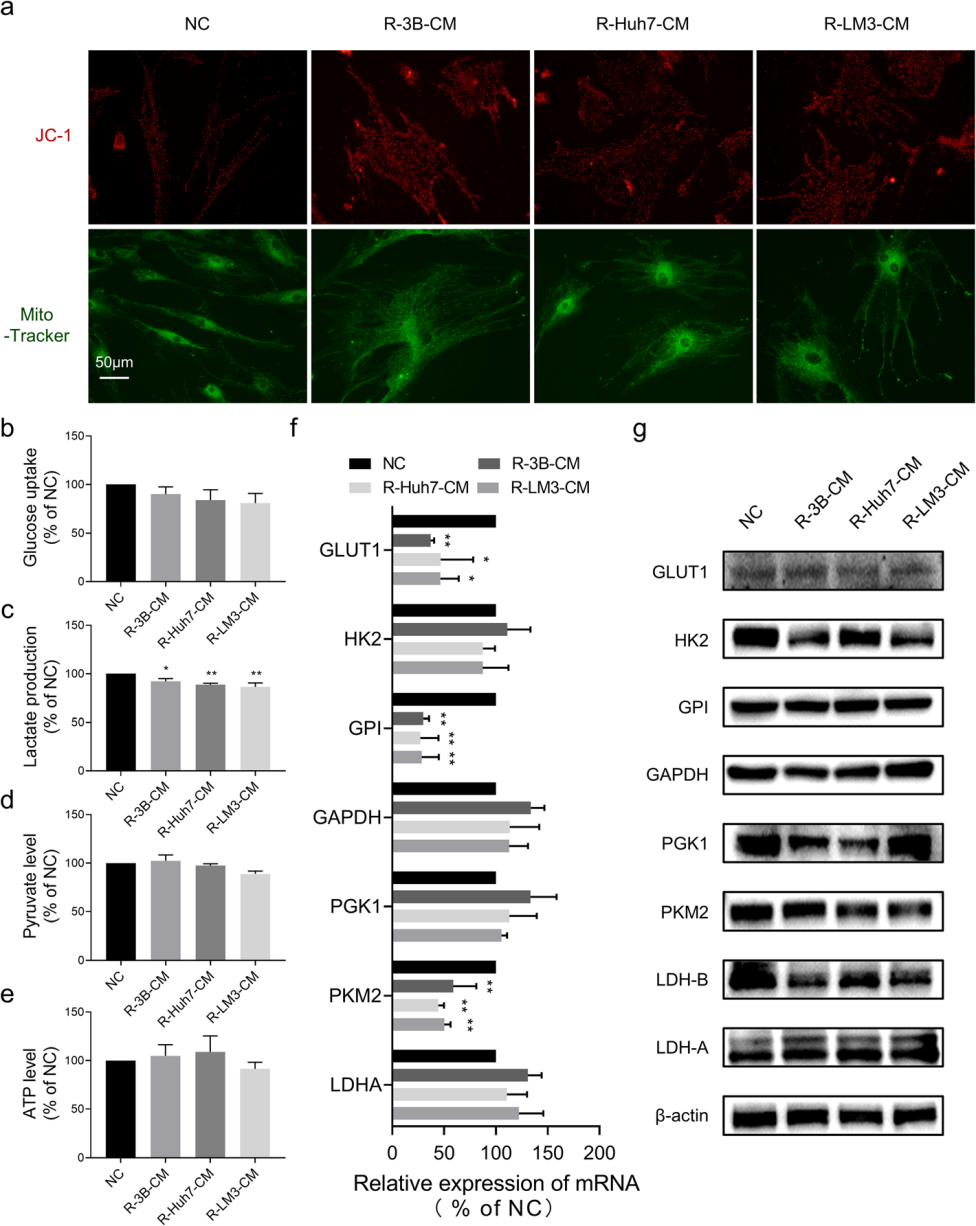
Glycolysis and mitochondrial function of hAT-MSCs after withdrawal of HCC-CM treatment. (5) Representative images of mitochondrial mass (Mito-Tracker) and MMP (JC-1) in hAT-MSCs after withdrawal of HCC-CM treatment; (**b**-**e**) Quantification of relative glucose uptake (**b**), lactate production (**c**), pyruvate level (**d**) and ATP level (**e**) in hAT-MSCs after withdrawal of HCC-CM treatment; (**f** & **g**) Analysis of glycolytic gene expression in hAT-MSCs after withdrawal of HCC-CM treatment. qRT-PCR (**f**) and immunoblot (**g**). *p < 0.05, **p < 0.01, vs NC, *n* = 3

As we found above, the mitochondrial apoptosis pathway of hAT-MSCs was reversed after withdrawal of HCC-CM treatment; thus, we wondered whether the function of mitochondria could also be reversed. Therefore, we used JC-1 and Mito-Tracker to stain mitochondria again. The staining results showed that the mitochondrial mass of hAT-MSCs was essentially unaffected after withdrawal of HCC-CM treatment, while there was a marked enhancement in MMP (Fig. 5a). These results suggest that HCC-CM-induced mitochondrial dysfunction was reversed.

Next, we tested the glycolytic phenotype in hAT-MSCs after withdrawal of HCC-CM treatment. The results showed a significant reduction in lactate production, but there were no significant differences in glucose uptake, pyruvate level and ATP level of hAT-MSCs after withdrawal of HCC-CM treatment (Fig. 5b-e). Moreover, after withdrawal of HCC-CM treatment, hAT-MSCs showed markedly reduced GLUT1, GPI and PKM2, but not HK2, GAPDH, PGK1 and LDHA, at the mRNA levels (Fig. 5f). At the protein levels, hAT-MSCs showed reduced levels of HK2, PGK1, PKM2 and LDHB (Fig. 5g).

These data indicate that the enhanced glycolysis in hAT-MSCs was reversed after withdrawal of HCC-CM treatment.

### HCC-CM regulated the activation of the ROS/MAPK/HIF-1α signaling pathway

**Fig. 6:**
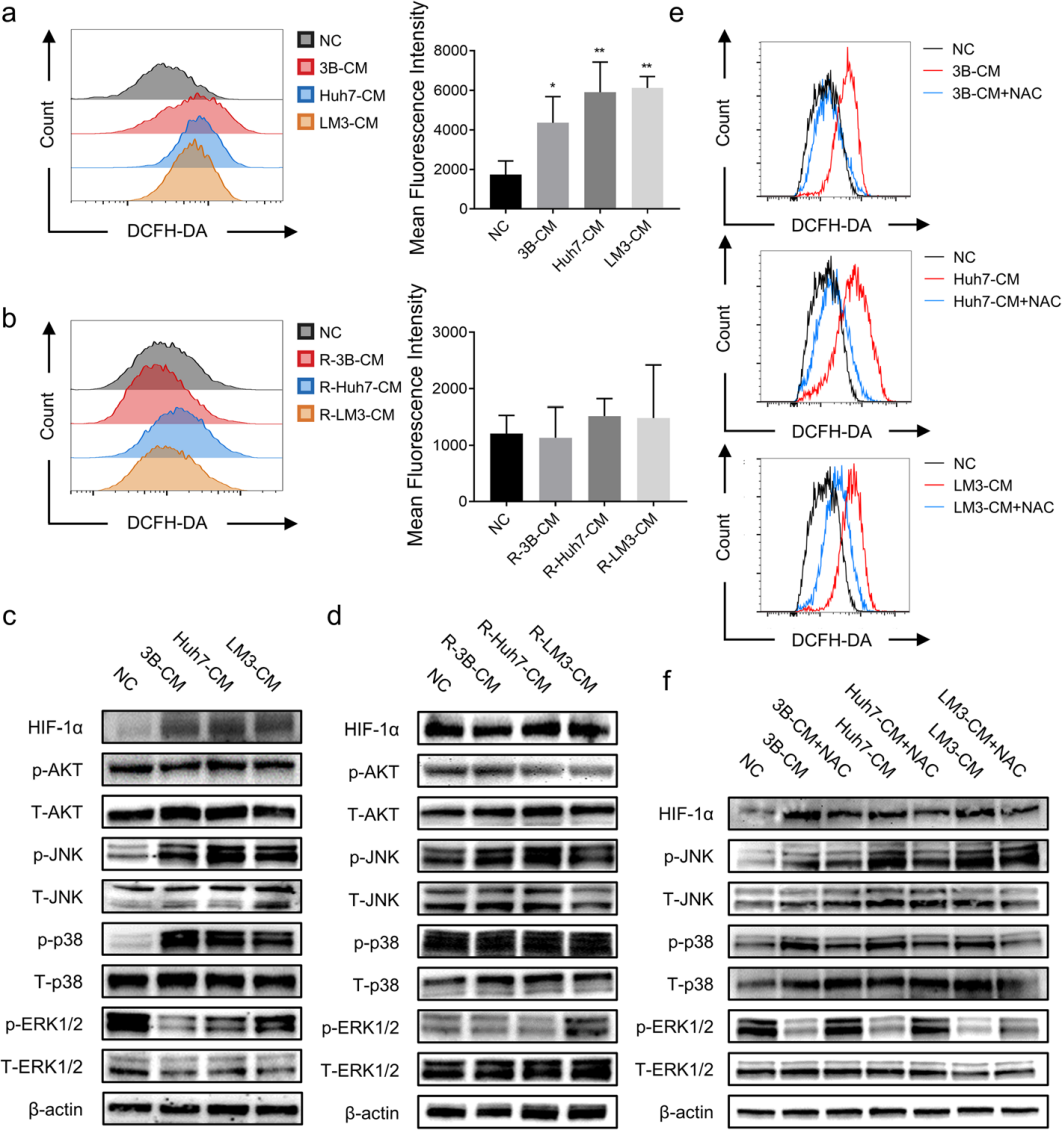
Effect of HCC-CM on the ROS/MAPK/HIF-1α signaling pathway. **a** Representative histogram (left) and quantification data (right) of ROS levels in hAT-MSCs after exposure to HCC-CM or normal medium; **b** Representative histogram (left) and quantification data (right) of ROS levels in hAT-MSCs after withdrawal of HCC-CM treatment; **c** HIF-1α, phosphorylated AKT, total AKT, phosphorylated JNK, total JNK, phosphorylated p38, total p38, phosphorylated ERK and total ERK protein expression of hAT-MSCs after exposure to HCC-CM or normal medium; **d** HIF-1α, phosphorylated AKT, total AKT, phosphorylated JNK, total JNK, phosphorylated p38, total p38, phosphorylated ERK and total ERK protein expression of hAT-MSCs after withdrawal of HCC-CM treatment; **e** ROS levels in hAT-MSCs after exposure to HCC-CM, normal medium or pre-treated with NAC; **f** HIF-1α, phosphorylated AKT, total AKT, phosphorylated JNK, total JNK, phosphorylated p38, total p38, phosphorylated ERK and total ERK protein expression of hAT-MSCs after exposure to HCC-CM, normal medium or pre-treated with NAC. *p < 0.05, **p < 0.01, vs NC, *n* = 2

Given the central roles that mitochondria played in phenotypic and metabolic alterations of hAT-MSCs, as mitochondria are the major source of ROS and since ROS can determine cell fate by regulating multiple signaling pathways, we used DCFH-DA probes to measure intracellular ROS. Our results showed that ROS levels significantly increased in hAT-MSCs after exposure to HCC-CM (Fig. 6a). However, after withdrawal of HCC-CM treatment, levels of ROS were restored to normal control levels (Fig. 6b).

To further investigate the possible roles of ROS-associated signaling pathways, we assessed the levels of HIF-1α, MAPK (ERK1/2, JNK and p38) and AKT. We found that exposure of HCC-CM resulted in activation of the HIF-1α and MAPK pathways, but not the AKT pathway. The phosphorylation levels of JNK and p38 were increased, while that of ERK1/2 was decreased (Fig. 6c). However, after withdrawal of HCC-CM treatment, the levels of HIF-1α and phosphorylated ERK, p38, and JNK were restored to normal control levels (Fig. 6d).

Next, to determine whether ROS are the upstream signal molecules of MAPK and HIF-1α pathways, we used the ROS scavenger NAC to block ROS generation. When cells were pre-treated with 5 mM NAC for 24 h, ROS production was severely suppressed (Fig. 6e). Meanwhile, the levels of HIF-1α and phosphorylated JNK and p38 were clearly reduced, and the phosphorylation level of ERK1/2 was restored (Fig. 6f). Besides, to elucidate the relationship between MAPK signaling pathway and cell phenotype, the treated hAT-MSCs were pre-treated with SB203580 (20 μM, 2 h), a kind of p38 inhibitor. We found that SB203580 could significantly rescue the loss of cell viability, including cell proliferation, cell apoptosis and MMP (Figure S1a-d).

These data suggest that the ROS/MAPK/HIF-1α signaling pathway plays an important role in HCC-CM-induced phenotypic and metabolic alterations of hAT-MSCs.

### HCC-CM endow hAT-MSCs with permanent secretory phenotype and tumor-promoting properties

**Fig. 7:**
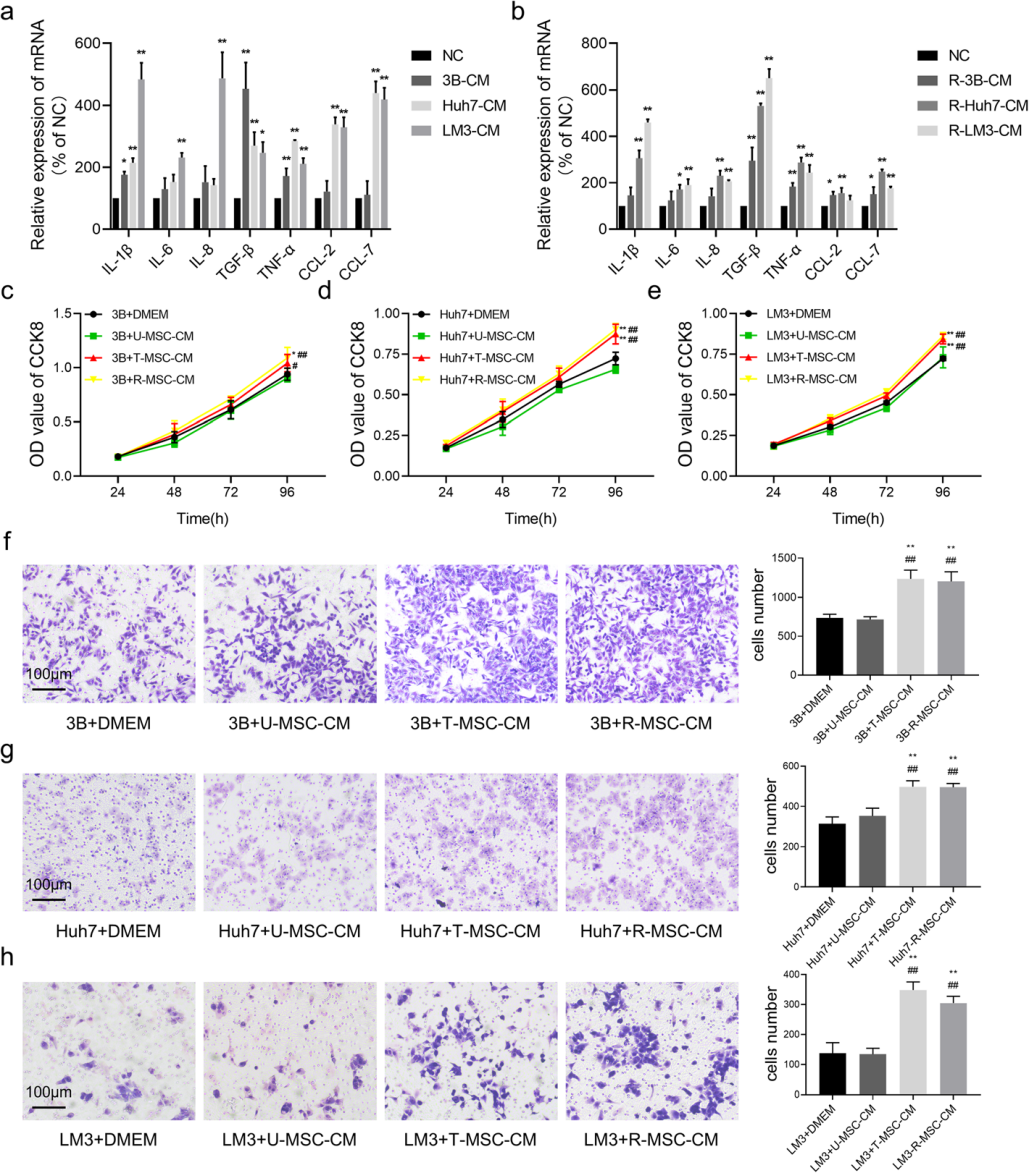
Secretory phenotype and tumor-promoting properties of hAT-MSCs after exposure to and withdrawal from HCC-CM. **a** & **b** mRNA expression of various factors in hAT-MSCs after exposure to (**a**) and withdrawal from HCC-CM (**b**) was determined by qRT-PCR; **c**-**e** Quantitative results of OD value at 570 nm in Hep3B (**c**), Huh7 (**d**) and HCCLM3 (**e**) after exposure to U-MSC-CM, T-MSC-CM, R-MSC-CM or normal medium; **f**-**h** Cell migration assays (left) and quantitative results (right, numbers of stained nuclei) of Hep3B (**f**), Huh7 (**g**) and HCCLM3 (**h**) after exposure to U-MSC-CM, T-MSC-CM, R-MSC-CM or normal medium. *p < 0.05, **p < 0.01, vs NC or DMEM, *#p* < 0.05, *##p* < 0.01, vs U-MSC-CM, *n* = 2, except for CCK-8 assays (*n* = 8)

The fate of hAT-MSCs had been seriously threatened after exposure to HCC-CM, however it can be reversed by withdrawal of HCC-CM treatment. We wondered whether these observations have functional relevance, then we investigated the effect of HCC-CM on secretory phenotype in hAT-MSCs, the mRNA expression of a variety of cytokines, growth factors and chemokines were analyzed. The expression levels of many factors, especially IL-1β, TGF-β and CCL-7, were much higher after exposure to HCC-CM than in untreated hAT-MSCs (Fig. 7a). and the characteristics of factors secretion by TA-MSCs appears to be permanent, since these cells keep producing these factors even after withdrawal of HCC-CM treatment (Fig. 7b).

We next determined whether the altered mRNA expression in TA-MSCs has functional relevance, CCK-8 and Transwell assays were used to investigate the effect of MSC-CM on proliferation and migration in HCC cells. No significant differences in proliferation and migration were observed when HCC cells were incubated with conditioned medium from untreated hAT-MSCs (U-MSC-CM), compared to the normal medium (DMEM) (Fig. 7c-h). In contrast, conditioned medium from treated hAT-MSCs (T-MSC-CM) and reversed hAT-MSCs (R-MSC-CM) significantly enhanced proliferation compared to DMEM and U-MSC-CM at 96-h point (Fig. 7c-e). Transwell migration assays demonstrated that T-MSC-CM and R-MSC-CM significantly increased the migration potency of HCC cells (Fig. 7f-h).

Taken together, these results revealed that HCC-CM endow hAT-MSCs with permanent tumor-promoting properties.

## Discussion

In this study, we have identified the regulation and reversal of hAT-MSC fate in an HCC-mimicking microenvironment. Our data indicate that in hAT-MSCs exposed to HCC-CM for 4–8 weeks, hAT-MSCs could evolve into TA-MSCs. Correspondingly, the phenotype and glucose metabolism of hAT-MSCs had changed dramatically. Moreover, for the first time, our results illustrated that after withdrawal of HCC-CM treatment for 2–4 weeks, the alterations of phenotype and glucose metabolism could be reversed and restored to normal, but secretory phenotype and tumor-promoting properties appear to be permanent. Importantly, we also revealed underlying mechanisms involving the promotion of ROS release and activation of the HIF-1α and MAPK signaling pathways.

Our experiments verified that mitochondria played core roles in phenotypic and metabolic alterations of hAT-MSCs, as represented here by activation of the mitochondrial apoptosis pathway and reduction of MMP, respectively. Mitochondria are cellular bioenergetic centres and major sources of ROS. However, under conditions of environmental stress, the overaccumulation of ROS can cause excessive oxidative stress and oxidant injury in mitochondria [15]. Then, the increase of mitochondrial damage can initiate the apoptosis cascade and result in cell death.

The disturbance in MMP is a main mark of mitochondrial dysfunction induced by oxidative stress. ROS can cause depolarization of MMP and activate the mitochondrial apoptosis pathway [16]. ROS can phosphorylate and activate JNK [17], which activates pro-apoptotic Bax, which can further inhibit the functions of anti-apoptotic Bcl-2. Inactivation of Bcl-2 can open channels on the outer mitochondrial membrane, which allows leakage of cytochrome c into the cytosol, eventually resulting in a cascade reaction of caspase to induce cell apoptosis [18]. The present work showed that levels of ROS underwent significant increases in hAT-MSCs with HCC-CM treatment, which consists with our data concerning a decline in MMP and activation of the JNK/mitochondrial apoptosis pathway. In addition, after withdrawal of HCC-CM treatment to reduce the oxidative stress, we found a significant reduction in intracellular ROS, which was accompanied by the reversal of phenotypic changes. Therefore, we have reason to speculate that the overaccumulation of intracellular ROS induced by environmental stress plays an important role in phenotypic changes, especially in cell apoptosis.

In addition to phenotypic changes of hAT-MSCs, the present work also addressed alterations in glucose metabolism. Our results showed that the decreased MMP was accompanied by enhanced glycolysis activity with HCC-CM treatment. Accumulating evidence indicates that mitochondrial function is dependent on the proton motive force, which is determined by the MMP. On the one hand, the overaccumulation of ROS can induce the decline of MMP and then disrupt electron transport and uncouple oxidative phosphorylation. To maintain an intracellular energy balance, cells must enhance glycolysis activity to compensate for the weakening of oxidative phosphorylation. On the other hand, ROS can trigger activation of HIF-1α, leading to hypoxia signaling and enhancing glycolysis [19]. These pathways are in line with the results observed in our study. To further explore the major driving force behind the switch in glucose metabolism, we studied the reversibility of mitochondrial function. The results showed that after withdrawal of HCC-CM treatment, mitochondrial dysfunction was reversed and glycolysis activity returned to normal levels. Based on this evidence, we speculate that reprogramming of glucose metabolism is an important protective mechanism for the regulation of cell fate. Under conditions of environmental stress, to reduce the production of ROS, cells actively switch their glucose metabolism from oxidative phosphorylation to aerobic glycolysis, thus improving their survival. Once they get rid of survival stress, cells can restore mitochondrial oxidative phosphorylation and efficient use of glucose to produce ATP.

We wondered whether reversibility of hAT-MSC phenotypic and metabolic changes have functional relevance, we further explore the secretory phenotype and tumor-promoting properties of treated and reversed hAT-MSCs. To our surprise, HCC-CM appears to endow hAT-MSCs with permanent secretory phenotype and tumor-promoting properties, since reversed hAT-MSCs exhibited a cytokine/chemokine expression pattern and tumor-promoting capability similar to treated hAT-MSCs. This indicates that the TME can induce permanent functional alterations during the conversion of MSCs into TA-MSCs, even if the phenotypes are reversed. Combined with the findings of our present study that hAT-MSCs underwent cytoskeleton remodeling and a significant increase in the migratory and invasion capacity with HCC-CM treatment, and the redistribution phenomenon of MSCs we mentioned above, all of these lines of evidence provide us with new ideas for studying TA-MSCs. We speculate that TA-MSCs are likely to play a “pioneer” role in tumor expansion and metastasis. Trapped in a stressed cage, MSCs have to remodel their cytoskeleton and continually invade the border between the tumor and normal tissue, thus improving their survival and changing their fate. Meanwhile, cells can keep exerting functions as TA-MSCs and constantly ‘remodel’ the tumor niche to further guide tumor progress, including the conversion of naïve MSCs to TA-MSCs and recruitment of macrophages to tumor sites [20, 21]. This will be the next research direction and focus.

Our study found that HCC-CM could significantly inhibit hAT-MSC proliferation, which was in contradiction with the previous studies. Numerous studies have demonstrated that MSCs obtained from tumor tissue exhibited higher growth capacity and proliferative activity [22]. Co-culture with cancer cells or induction by conditioned medium in vitro also resulted in similar findings [8]. We speculate that one reason for this inconsistency was different induction times in vitro. No matter what kind of induction manner is adopted, the action time was often no longer than 2 weeks in past studies. We hold the opinion that the induction time was generally too short to simulate long-term changes of cells in the body. Therefore, we extended HCC-CM treatment for 4–8 weeks. The reason why we adopted the different treatment length with HCC-CM is that due to the time costs considerations. We need to ensure that the treatment time is long enough, meanwhile minimize the effects of MSC aging on cell phenotype. The proliferation of hAT-MSC is exceptionally slow, and it will take more than a week for one passage. Therefore, 4-week treatment will not excessively increase passage number of cells and result in cell senescence. At the same time, after 4-week treatment, phenotypic and metabolic changes of hAT-MSCs are very stable, we have analyzed at different time point, the results were consistent. In addition, treating the same time in each of the independent experiments will also minimize the variable. However, since we did not dynamically monitor the changes of proliferation in hAT-MSCs, it is likely to ignore the possibility of enhanced proliferation in the early stage of induction, or it could be caused by neglecting the reversibility of the phenotype. Choosing MSCs derived from tumor tissue as the research object can solve the problem of insufficient induction time; however, by using normal medium for cell expansion in vitro, the reversal of the cell phenotype is very likely to distort the results. In our study, although there was no significant increase in the proliferation capacity of hAT-MSCs after withdrawal of HCC-CM treatment, it did increase in value.

Overall, the current study confirmed that HCC-CM can regulate the phenotype and glucose metabolism of hAT-MSCs through the ROS/MAPK/HIF-1α signaling pathway. In addition, through analysis of our data, we believe that the accumulation of ROS caused by environmental stress led to the activation of the MAPK/HIF-1α signaling pathway, which is the key to changes in hAT-MSCs. If antioxidants or genetic engineering are used to transform MSCs and improve their antioxidant capacity, MSCs may improve their survival in the tumor environment and become better tools for drug delivery. Moreover, we demonstrated the reversibility of phenotype and glucose metabolism in hAT-MSCs, which is critically important for generating novel ideas for TA-MSC research. However, our study was not designed to identify which components present in HCC-CM play roles. Thus, we cannot perform selective and precise targeting of the substance to block phenotypic and metabolic changes of hAT-MSCs. Further experiments are clearly necessary.

## Conclusions

In conclusion, the effects of long-term HCC-CM treatment on phenotype and glucose metabolism in hAT-MSCs are modest and largely reversible after withdrawal, but HCC-CM endow hAT-MSCs with permanent secretory phenotype and tumor-promoting properties. And these alterations were achieved through regulation of the ROS/MAPK/HIF-1α signaling pathway. This is the first report on the reversal of phenotype and glucose metabolism in TA-MSCs, and it might provide new insights of TA-MSCs to develop potential strategies for MSC-based anti-tumor therapy.

## Supplementary Information

**Additional file 1:** **Figure S1.** Effect of SB203580 on cell phenotype.

## Data Availability

The datasets generated/analyzed during the current study are available.

## Abbreviations

hAT-MSCs: Human adipose tissue-derived mesenchymal stem cells
HCC: Hepatocellular carcinoma
CM: Conditioned medium
TME: Tumor microenvironment
CCK-8: Cell counting kit-8
ROS: Reactive oxygen species
MAPK: Mitogen activated protein kinase
HIF-1α: Hypoxia inducible factor 1 alpha
CAFs: Cancer-associated fibroblasts
TA-MSCs: Tumor-associated mesenchymal stem cells
αSMA: α-smooth muscle actin
CD: Cluster of differentiation
PBS: Phosphate buffered saline
DMEM: Dulbecco’s Modified Eagle’s medium
FBS: Fetal bovine serum
DNA: Deoxyribonucleic acid
RNA: Ribonucleic acid
RT-qPCR: Real time quantitative polymerase chain reaction
ATP: Adenosine triphosphate
BSA: Bovine serum albumin
PMSF: Phenylmethanesulfonylfluoride
MMP: Mitochondrial membrane potential
3B-CM: Hep3B-conditioned medium
Huh7-CM: Huh7-conditioned medium
LM3-CM: HCCLM3-conditioned medium
R-3B-CM: Reversed-Hep3B-conditioned medium
R-Huh7-CM: Reversed-Huh7-conditioned medium
R-LM3-CM: Reversed-HCCLM3-conditioned medium
U-MSC-CM: Untreated hAT-MSC conditioned medium
T-MSC-CM: Treated hAT-MSC conditioned medium
R-MSC-CM: Reversed hAT-MSC conditioned medium
