# Supplementary material for: Reversibility of hAT-MSCs phenotypic and metabolic changes after exposure to and withdrawal from HCC-conditioned medium through regulation of the ROS/MAPK/HIF-1α signaling pathway
Source: Stem Cell Res Ther. 2020 Nov 27;11:506. doi: 10.1186/s13287-020-02010-0 (PMC7694319; doi:10.1186/s13287-020-02010-0)
Supplement: Supplementary file 1 — Additional file 1: Figure S1. Effect of SB203580 on cell phenotype. [file 13287_2020_2010_MOESM1_ESM.zip › Supplement.docx]

**Reversibility of hAT-MSCs phenotypic and metabolic changes after** **exposure to and** **withdrawal form HCC-conditioned medium** **through regulation of the ROS/MAPK/HIF-1α signalling pathway**

**Figure S1 Effect of SB203580 on cell phenotype.** (a) Phosphorylated p38 and total p38 protein expression of the treated hAT-MSCs after being pre-treated with SB203580; (b) Quantitative results of OD value at 570 nm in the treated hAT-MSCs after being pre-treated with SB203580; (c) Representative images (left) and quantification results (right) of cell apoptosis in the treated hAT-MSCs after being pre-treated with SB203580; (d) Representative images of mitochondrial mass (Mito-Tracker, 100nM) and MMP (JC-1, 10μg/ml) in the treated hAT-MSCs after being pre-treated with SB203580. *##p*<0.05, vs 3B-CM, ***p*<0.01, vs NC.
