# Supplementary figures and images for: Reversibility of hAT-MSCs phenotypic and metabolic changes after exposure to and withdrawal from HCC-conditioned medium through regulation of the ROS/MAPK/HIF-1α signaling pathway
Source: Stem Cell Res Ther. 2020 Nov 27;11:506. doi: 10.1186/s13287-020-02010-0 (PMC7694319; doi:10.1186/s13287-020-02010-0)

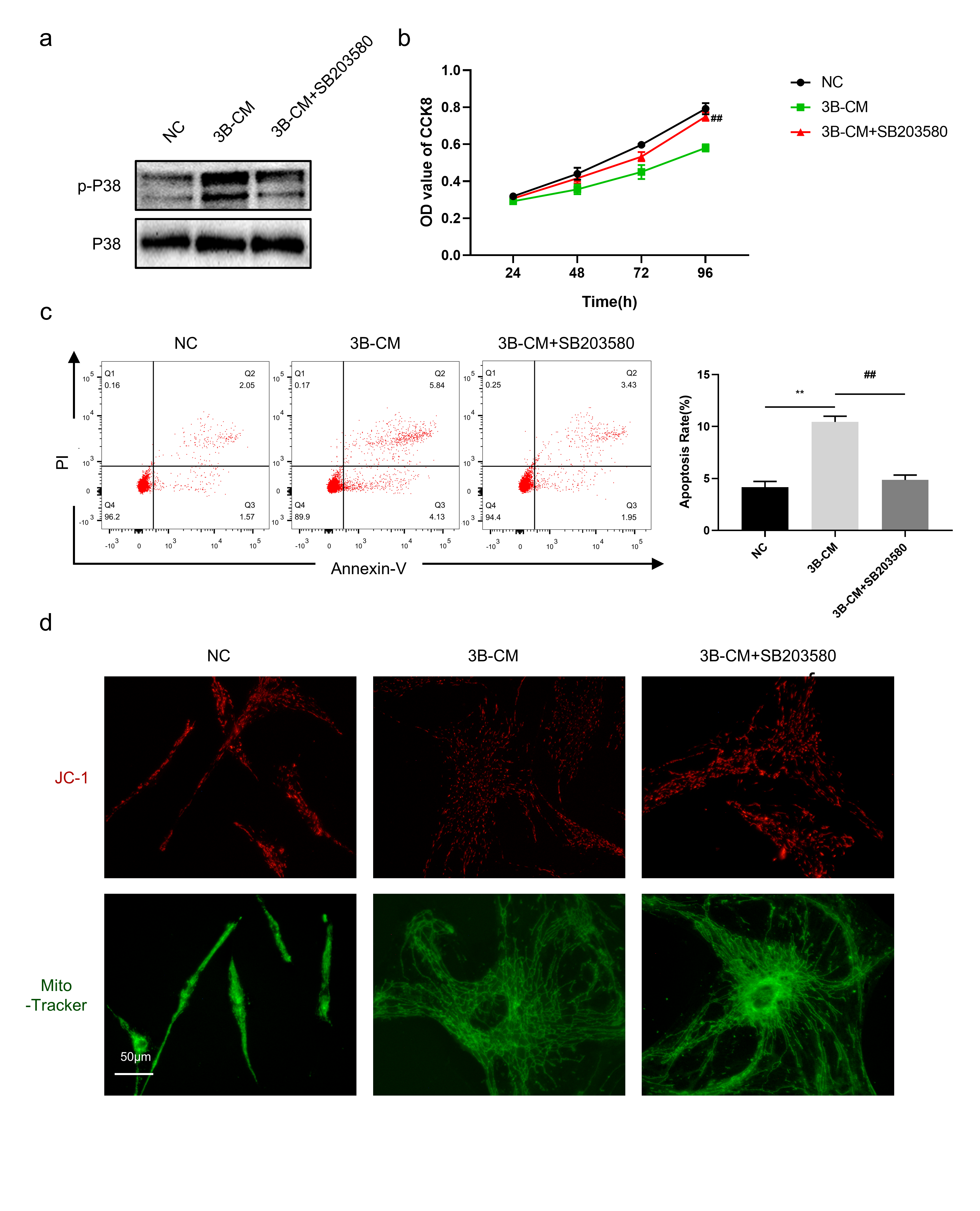

Supplement: Supplementary file 1 — Additional file 1: Figure S1. Effect of SB203580 on cell phenotype. [file 13287_2020_2010_MOESM1_ESM.zip › figure S1.tif]
